# Prophylactic Efficacy of TcVac2 against *Trypanosoma cruzi* in Mice

**DOI:** 10.1371/journal.pntd.0000797

**Published:** 2010-08-10

**Authors:** Shivali Gupta, Nisha Jain Garg

**Affiliations:** 1 Departments of Microbiology and Immunology, University of Texas Medical Branch, Galveston, Texas, United States of America; 2 Department of Pathology, University of Texas Medical Branch, Galveston, Texas, United States of America; 3 Member of the Institute for Infections and Immunity, Center for Biodefense and Emerging Infectious Diseases, and Sealy Center for Vaccine Development, University of Texas Medical Branch, Galveston, Texas, United States of America; University of Massachusetts Medical School, United States of America

## Abstract

**Background:**

Chagas disease is a major health problem in Latin America, and an emerging infectious disease in the US. Previously, we have screened the *Trypanosoma cruzi* sequence database by a computational/bioinformatics approach, and identified antigens that exhibited the characteristics of vaccine candidates.

**Methodology:**

We investigated the protective efficacy of a multi-component DNA-prime/protein-boost vaccine (TcVac2) constituted of the selected candidates and cytokine (IL-12 and GM-CSF) expression plasmids in a murine model. C57BL/6 mice were immunized with antigen-encoding plasmids plus cytokine adjuvants, followed by recombinant proteins; and two-weeks later, challenged with *T. cruzi* trypomastigotes. ELISA and flow cytometry were employed to measure humoral (antibody isotypes) and cellular (lymphocyte proliferation, CD4^+^ and CD8^+^ T cell phenotype and cytokines) responses. Myocardial pathology was evaluated by H&E and Masson's trichrome staining.

**Principal Findings:**

TcVac2 induced a strong antigen-specific antibody response (IgG2b>IgG1) and a moderate level of lymphocyte proliferation in mice. Upon challenge infection, TcVac2-vaccinated mice expanded the IgG2b/IgG1 antibodies and elicited a substantial CD8^+^ T cell response associated with type 1 cytokines (IFN-γ and TNF-α) that resulted in control of acute parasite burden. During chronic phase, antibody response persisted, splenic activation of CD8^+^ T cells and IFN-γ/TNF-α cytokines subsided, and IL-4/IL-10 cytokines became dominant in vaccinated mice. The tissue parasitism, inflammation, and fibrosis in heart and skeletal muscle of TcVac2-vaccinated chronic mice were undetectable by histological techniques. In comparison, mice injected with vector or cytokines only responded to *T. cruzi* by elicitation of a mixed (type 1/type 2) antibody, T cell and cytokine response, and exhibited persistent parasite burden and immunopathology in the myocardium.

**Conclusion:**

TcVac2-induced activation of type 1 antibody and lymphocyte responses provided resistance to acute *T. cruzi* infection, and consequently, prevented the evolution of chronic immunopathology associated with parasite persistence in chagasic hearts.

## Author Summary

*Trypanosoma cruzi*, a parasitic protozoan, is the etiologic agent of Chagas disease. Chagas disease is the most common cause of congestive heart failure related deaths among young adults in the endemic areas of South and Central America and Mexico. Vaccine development against Chagas disease has been dramatically limited because of extensive debate on the mechanisms involved in this pathology. It is now accepted that the presence of parasites in cardiac tissue is necessary to initiate and maintain the inflammatory responses and that therapeutic treatments or vaccines aimed at eliminating *T. cruzi* would limit or prevent the progression of chronic chagasic cardiomyopathy. In the present study, we have tested the protective efficacy of a multi-component heterologous DNA-prime/protein-boost vaccine TcVac2 in a murine model of *T. cruzi* infection. Immunization of mice with TcVac2 induced potent antibody, CD8^+^ T cell and cytokine responses that provided protection from acute parasitemia and chronic parasite persistence and immunopathology in chagasic mice in comparison to unvaccinated mice.

## Introduction 

*Trypanosoma cruzi*, a parasitic protozoan, is the etiologic agent of Chagas disease. Chagas disease is the most common cause of congestive heart failure related deaths among young adults in the endemic areas of South and Central America and Mexico [1]. It has also become an important health issue in the United States and Europe due to large scale migration of Latin Americans over the last few decades [2]. No vaccines are currently available.

Several investigators have documented that protective immune mechanisms against *T. cruzi* are constituted of a strong lytic antibody response, cytotoxic T lymphocytes activity, and Th1 cytokines [3]–[7] Towards identifying the potential vaccine candidates, *T. cruzi* transfectants expressing ovalbumin (OVA, model antigen) in different cellular compartments were developed. Immunological studies in mice infected with OVA-transfectants suggested that parasite GPI-anchored (released by default in host cell cytoplasm) and secreted proteins were capable of entering the class I and class II pathways of antigen presentation and eliciting antibody and T cell responses, and, thus, would be the best choice as vaccine candidates [8]. Accordingly, several GPI-anchored proteins of *T. cruzi* have been identified, and their immunogenic potential examined in mice. Many of the selected antigenic targets provided variable degree of resistance to *T. cruzi* as DNA or protein vaccine [5], [9]–[13] (reviewed in [14]–[16]).

In parallel with the efforts towards identification of vaccine [9] candidates, the development of methods to enhance the protective efficacy of subunit vaccines against *T. cruzi* has been the primary focus of research for several years. These include the use of adjuvants, e.g. saponin with GP90 [17], alum with GP82 [9], IL-12 and CpGODN with cruzipain [12] [18], [19] and IL-12 and GMCSF in numerous studies (reviewed in [20]). Others have used attenuated strain of Salmonella [21] adenovirus [22], [23] for antigen delivery, or a heterologous prime-boost protocols [24], [25] to enhance the selected candidates efficacy against *T. cruzi*.

We have employed an unbiased computational/bioinformatics approach for screening the *T. cruzi* sequence database and identification of potential vaccine candidates [26]. A strategic analysis of the sequence database led to selection of 71 candidates that were unique to *T. cruzi*, but not the members of the trans-sialidase, mucin or other large gene families, and exhibited multiple motifs/characteristics of secreted or GPI-anchored proteins. Of these, eight candidates (TcG1, TcG2, TcG3, TcG4, TcG5, TcG6, TcG7, and TcG8 [TcG1-TcG8]) were phylogenetically conserved in clinically important strains of *T. cruzi*, expressed in the infective and intracellular stages of the parasite [26], and elicited varying level of lytic antibody response and Th1 cytokines (IFN-γ), a property associated with immune control of *T. cruzi,* in immunized mice. TcG1-, TcG2-, and TcG4-encoded antigens were expressed on the plasma membrane of the mammalian stages of *T. cruzi* (trypomastigote/amastigote) and elicited significant levels of anti-parasite lytic antibody responses in mice [26]. These novel vaccine candidates, thus, increased the pool of protective vaccine [17] candidates against *T. cruzi*.

Heterologous DNA-prime and protein-boost is a promising vaccination approach. Delivery of antigens as DNA vaccine elicits robust T cell response that can be further enhanced and modulated to type 1 by co-delivery of IL-12 and GMCSF cytokine adjuvants [27], [28]. DNA immunization is also effective in priming antigen-specific memory B cells. Delivery of vaccine candidates as recombinant proteins is generally more effective at eliciting antibody responses and may directly stimulate antigen-specific memory B cells to differentiate into antibody-secreting cells, resulting in production of high titer antigen-specific antibodies [29], [30]. Therefore, DNA-prime plus protein-boost is a complementary approach that overcomes the immunological limitations of DNA/DNA and protein/protein vaccines, and DNA/protein vaccines have been shown to elicit stronger, long-term cellular immunity against intracellular pathogens [31], [32].

In this study, we tested the protective efficacy of a DNA-prime/protein-boost subunit vaccine (TcVac2) constituted of candidate antigens that were identified by the computational/bioinformatic analysis of *T. cruzi* sequence database. We discuss the TcVac2 efficacy in gearing the antibody, T cell and cytokine responses that provided protection from acute parasitemia, and chronic parasite persistence and immunopathology in chagasic mice.

## Methods

### Parasites and mice

Trypomastigotes of *T. cruzi* (Sylvio X10/4 strain) were maintained and propagated by continuous *in vitro* passage in C2C12 cells. C57BL/6 female mice (6-to-8 weeks old) were obtained from Harlan Labs (Indianapolis, IN). Animal experiments were performed according to the National Institutes of Health Guide for Care and Use of Experimental Animals and approved by the UTMB Animal Care and Use Committee.

### *T. cruzi* genes, plasmid construction, and recombinant proteins

Sequences for TcG1, TcG2 and TcG4 have previously been submitted to Genbank (AY727914, AY727915, and AY727917, respectively). TcG1 is 76% identical to the *Leishmania donovani* 23-kDa cell surface protein [33]. Tc*G2* and TcG4 have been identified in CL Brenner sequence database, and exhibit 99–100% homology to XM_806323 and XM_816508, respectively [34].

The cDNAs for *TcG1, TcG2,* and *TcG4* were cloned in eukaryotic expression plasmid pCDNA3.1 [26] for DNA vaccination purposes. The eukaryotic expression plasmids encoding murine IL-12 (pcDNA3.msp35 and pcDNA3.msp40) and GM-CSF (pCMVI.GM-CSF) cytokines have been previously described [5]. Recombinant plasmids were transformed into *E. coli* DH5-alpha competent cells, grown in L-broth containing 100 µg/ml ampicillin, and purified by anion exchange chromatography using the Endo free maxi prep kit (Qiagen, Valencia, CA), according to the manufacturer's specifications.

For protein vaccination, cDNAs for *TcG1, TcG2,* and *TcG4* were cloned in pET-22b plasmid (Novagen, Gibbstown, NJ) such that the encoded proteins were in-frame with a C-terminal His_5_-tag. For the purification of recombinant proteins, plasmids were transformed in BL21 (DE3) pLysS competent cells (Invitrogen, Carlsbad CA) and recombinant proteins purified using the poly-histidine fusion peptide-metal chelation chromatography system (Novagen). After purification, proteins were exchanged out of elution buffer by dialysis, and we validated that LPS contamination in the proteins was <1.0 EU/ml determined by toxin sensor limulus amebocyte lysate (LAL) assay kit (Genscript, USA Inc.). All cloned sequences were confirmed by restriction digestion and sequencing at the Recombinant DNA Core Facility at UTMB.

### Immunization and challenge infection

C57BL/6 mice were injected in the quadriceps muscle twice at three-week intervals with antigen-encoding plasmids (pCDNA3.TcG1, pCDNA3.TcG2 and pCDNA3.TcG4, 25 µg each plasmid DNA/mouse) and cytokine-encoding plasmids (pcDNA3.msp35, pcDNA3.msp40 (IL-12) and pCMVI.GM-CSF, 25 µg each plasmid DNA/mouse). Mice were then immunized with two doses of TcG1-, TcG2-, and TcG4- recombinant proteins (25 µg each, total 75 µg protein emulsified in 5 µg saponin/100 µl PBS/mouse, intra-dermal). Two weeks after the last immunization, mice were challenged with *T. cruzi* (10,000 trypomastigotes/mouse, i.p.). Mice were sacrificed at day 30 and 120 post-infection (pi) corresponding to the acute phase of peak parasitemia and the chronic phase of disease development, respectively. Sera and tissue samples (heart and skeletal muscle) were harvested, and stored at 4°C and −80°C, respectively.

### Antibody response

Sera samples were analyzed for IgG antibody levels by use of an Enzyme Linked Immunosorbent Assay (ELISA) [13]. *T. cruzi* lysate (5×10^5^ parasites equivalent/well) or recombinant antigens (20 µg/ml) were used to capture the *T. cruzi-* and antigen-specific antibodies, respectively. To identify the antibody sub-types, plates were coated with *T. cruzi* antigen, and, then sequentially incubated at room temperature with sera samples (1∶50–1∶1000 dilution, 100 µl/well) for 2 h, biotin-conjugated goat anti-mouse Ig subtypes (IgG1, IgG2a, or IgG2b) for 30 min, and streptavidin-horseradish peroxidase conjugate for 30 min. All antibodies and conjugates were from Southern Biotech, and used at a 1∶5000 dilution in PBST-0.5% NFDM (100-µl/well). Color was developed with 100-µl/well Sure Blue TMB substrate (Kirkegaard & Perry Labs, Gaithersburg, MD), reaction was stopped with 2N sulfuric acid, and antibody response was monitored at 450 nm using a SpectraMax M5 microplate reader. End point titers were defined as the highest serum dilution that resulted in an O.D. value greater than that of the mean plus two standard deviations of control mouse serum.

### Lymphocyte proliferation and cytokine levels

*T. cruzi* trypomastigote lysate (TcTL) was prepared by subjecting parasites (10^9^/ml PBS) to six cycles of repeated freezing and thawing followed by sonication in an ice-cold water bath for 30 min. Single-cell splenocyte preparation from immunized and control mice was suspended in RPMI-5% FBS. Cells were adjusted to a concentration of 5×10^5^ cells/ml and were cultured in 24- or 96-well plates for 48–72 h with added recombinant proteins (10-µg/ml), *T. cruzi* lysate (25-µg/ml) or concanavalin A (Con A, 5 µg/ml). Culture supernatants were collected at 48 h for the measurement of IFN-γ, TNF-α, IL-4 and IL-10 cytokines using optEIA^tm^ ELISA kits (Pharmingen, San Diego, CA), according to the manufacturer's specifications. The cell suspension in 96-well plates was stimulated for up to 72 h and utilized to measure the rate of lymphocyte proliferation by MTT assay [35]. In this assay, the yellow tetrazolium salt (3-(4,5-dimethylthiazol-2-yl)-2,5-diphenyltetrazolium bromide) is reduced by mitochondrial dehydrogenases in metabolically active live cells to insoluble purple formazan crystals that are solubilized and quantitated at 540 nm by spectrophotometry.

Splenic level of CD4^+^ and CD8^+^ cell population in immunized/challenged mice was determined by flow cytometry. Briefly, splenocytes were suspended in PBS (1×10^6^ cells/100 µl) and incubated for 30 min with FITC-conjugated anti-CD8 and PE-conjugated anti-CD4 antibodies (1∶50 dilution) (e-Biosciences, CA). Following incubation, cells were fixed with 2% paraformaldehyde, washed and re-suspended in 500 µl PBS, and analyzed on a FACScan apparatus (BD Biosciences). Cells stained with PE- and FITC- conjugated rat IgGs (isotype matched) were used as negative controls. Flow data were analyzed by Cell Quest software (BD Biosciences).

### Tissue parasite burden and histopathology

Skeletal muscle, heart spleen, liver and kidney tissues (50 mg) were subjected to Proteinase K lysis, and total DNA was purified by phenol/chloroform extraction and ethanol precipitation method. Total DNA (100 ng) isolated from heart and skeletal muscle was used as a template in a PCR reaction for 28 cycles with oligonucleotides specific for *T. cruzi* 18S ribosomal DNA sequence (Forward: 5′-TAGTCATATGCTTGTTTC-3′, Reverse: 5′-GCAACAGCATTAATATACGC-3′) [36]. For validation of PCR results, parasite burden in all tissues was monitored by real-time PCR on an iCycler thermal cycler (Bio-Rad) with SYBR Green Supermix (Bio-Rad) and Tc18S-sepecific oligonucleotides. The real-time PCR amplification of *Tc18SrDNA* was calculated using the formula fold change  = 2^−ΔCt^, where ΔC_t_ represents the C_t_ (infected sample) - C_t_ (control) [37]. All data were normalized with host-specific GAPDH.

For histological studies, skeletal muscle and heart tissues were fixed in 10% buffered formalin for 24 h, dehydrated in absolute ethanol, cleared in xylene, and embedded in paraffin. Five-micron tissue sections were stained with hematoxylin and eosin or Masson's Trichrome, and evaluated by light microscopy. Tissues were examined for evidence of cellular inflammation and fibrosis and each section was assigned a histological score. The presence of inflammatory cells was scored as (0) - absent/none, (1) - focal or mild myocarditis with ≤1 foci, (2) - moderate with ≥2 inflammatory foci, (3) - extensive with generalized coalescing of inflammatory foci or disseminated inflammation with minimal necrosis and retention of tissue integrity, and (4) - severe with diffused inflammation, interstitial edema, and loss of tissue integrity. The foci of fibrosis and pseudocysts (parasite nests) were scored as (0) absent, (1) 0–1 foci, (2) 1–5 foci, and (3)>5 foci.

### Statistical analysis

Data are expressed as means ± SD, and derived from at least triplicate observations per sample (n≥8 animals/group). Results were analyzed for significant differences using ANOVA procedures and Student's t-tests. The level of significance was calculated vector only-versus-immunized and cytokine adjuvants only-versus-vaccinated with TcVac2, and presented as ^#^ *p*<0.05, ^##^ *p*<0.01, ^###^ *p*<0.001.

## Results

**Figure 1:**
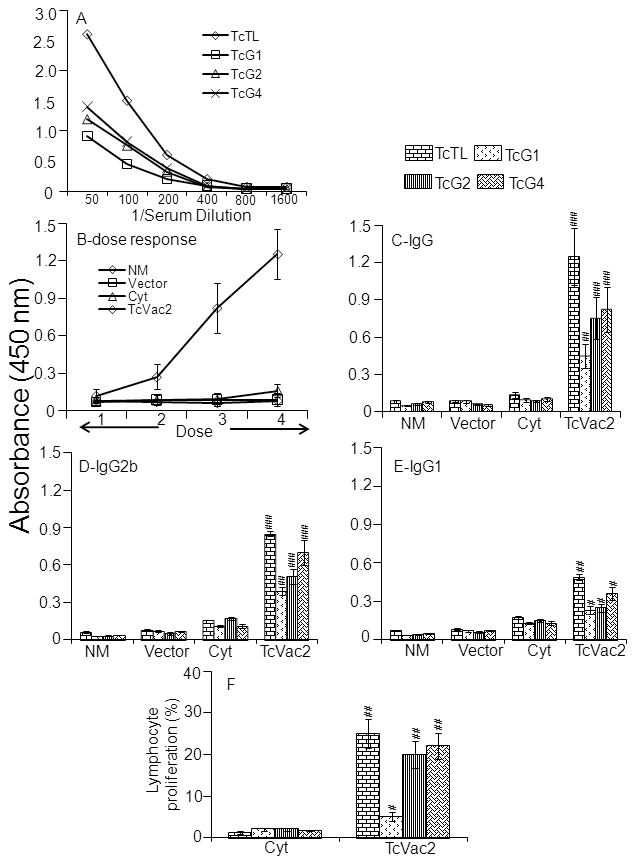
TcVac2 induced antigen-specific antibody response and lymphocyte proliferation in immunized mice. C57BL/6 mice were immunized with TcVac2 as detailed in Materials and Methods and sera samples were collected after each immunization. (**A**) Sera samples after last immunization were used to generate titration curves for *T. cruzi* (TcTL) and antigen-specific (TcG1, TcG2, TcG4) antibody detection in TcVac2-vaccinated mice. (**B**) *T. cruzi* specific IgG antibodies (sera dilution, 1∶100) were exponentially increased after each dose of immunization in vaccinated mice. (**C–E**) Sera levels of parasite- and antigen-specific IgG (** ***C*** **), IgG2b (** ***D*** **) and IgG1 (** ***E*** **) antibodies, measured two-weeks after the last immunization. Sera levels of antibody isotypes in normal mice (NM), and mice injected with vector alone or cytokine (Cyt) adjuvants only are also shown. (**F**) Spleen cells were obtained 2-weeks after last immunization and *in vitro* stimulated for 48 h with *T. cruzi* trypomastigotes lysate (TcTL) or recombinant antigens (TcG1, TcG2 and TcG4). Shown are the extent of lymphocytes proliferation in TcVac2-vaccinated mice and mice given cytokines only (data normalized to control mice given vector alone), measured by an MTT assay. Data (mean **±** SD) are representative of three independent experiments (n = 8 mice/group, ^#^ *p*<0.05, ^##^ *p*<0.01, ^###^ *p*<0.001).

Sera samples were collected after each immunization, and we determined the development of TcVac2-induced antibody response by an ELISA. Titration curves with sera after 4^th^ immunization indicated that sera dilution at 1∶100 provides the highest signal-to-noise ratio and ability to detect antibody response in linear range (Fig. 1A). All experiments were, therefore, conducted using sera dilution of 1∶100. Our data showed the sera level of *T. cruzi-*specific antibodies were detectable after the second immunization, and increased following each booster immunization with recombinant proteins (Fig. 1B). Antigen-specificity of antibody response, determined in sera collected after last immunization, is shown in Fig. 1C–E. We detected a substantial level of TcTL- and TcG1-, TcG2- and TcG4-specific IgG (Fig. 1C), IgG2b (Fig. 1D), and IgG1 (Fig. 1E) antibodies in mice immunized with TcVac2 (all, *p*<0.01−0.001). The level of antigen-specific antibody response was detected in the order of TcG4>TcG2>TcG1; and the antigen-specific antibody response was predominantly of the Th1 type with IgG2b/IgG1 ratio>1 (Fig. 1D–E). Control mice immunized with plasmid vector alone or cytokine adjuvants only exhibited no parasite-(TcTL) and antigen-specific (TcG1, TcG2 TcG4) antibody response (Fig. 1C–E).

To determine if vaccination elicited cellular responses, spleen cells were collected two-weeks after last immunization, *in vitro* stimulated with TcTL or recombinant proteins, and lymphocytes' proliferation and cytokine secretion assessed. Mice vaccinated with TcVac2 exhibited up to 25% increase in TcTL- and antigen-specific (TcG4 = TcG2>TcG1) lymphocytes' proliferation (Fig. 1F, *p*<0.05−0.001). Mice injected with cytokine adjuvants or vector only exhibited minimal to no TcTL- and antigen-specific lymphocytes' proliferation (Fig. 1F). The cytokine levels (IFN-γ, TNF-α and IL-10) were below detection limit in supernatants collected after 48 h *in vitro* stimulation of splenocytes from vaccinated and non-vaccinated mice. Incubation with Con A resulted in a substantial increase in IFN-γ, TNF-α and IL-10 levels in splenocytes from all animal groups (range: 800**±**65.8−2500**±**215 pg/ml). Together, these results demonstrated that TcVac2 induced TcTL- and antigen-specific Th1 antibodies, and lymphocytes' proliferation in immunized mice.

**Figure 2:**
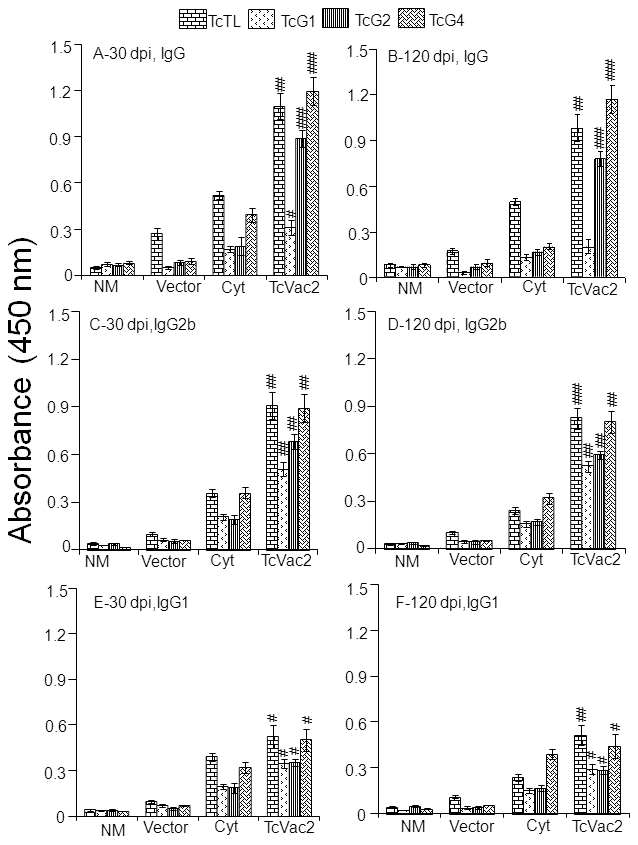
Antigen-specific antibodies were enhanced in response to challenge *T. cruzi* infection in TcVac2-immunized mice. Mice were vaccinated with TcVac2 and two-weeks after last immunization, infected with *T. cruzi* (10,000 trypomastigotes / mouse). Sera were collected at day 30 (**A,C&E**) and day 120 (**B,D&F**) post-infection. The sera level of *T. cruzi*- and antigen-specific IgG (**A&B**), IgG2b (**C&D**) and IgG1 (**E&F**) antibodies were measured by an ELISA (n = 8 mice/group, ^#^ *p*<0.05, ^##^ *p*<0.01,^ ###^ *p*<0.001). Abbreviations are as in Figure 1.

To determine if vaccination with TcVac2 primed an improved immune response to challenge infection, mice were infected with *T. cruzi* and antibody response, lymphocytes' proliferation and cytokine levels monitored at the acute (30 dpi) and chronic (120 dpi) stages of infection and disease development. Shown in Figure 2 are antibody levels in vaccinated/challenged mice (sera dilution, 1∶1000), determined by an ELISA. Vaccinated mice exhibited a rapid increase in antibody response upon challenge infection as was evidenced by ∼2–3-fold higher levels of parasite- and antigen-specific IgGs in vaccinated/acutely-infected mice as compared to non-vaccinated/infected mice (Fig. 2A, *p*<0.05−0.001). The higher levels of TcTL- and antigen-specific antibody response in vaccinated mice were maintained during the chronic phase (Fig. 2B, *p*<0.05−0.001). Similarly, sera level of IgG2b/IgG1 antibodies in TcVac2-vaccinated mice were higher than that noted in unvaccinated mice at all stages of infection and disease development (Fig. 2C–2F, *p*<0.05−0.001). The level of antigen-specific IgG and IgG2b antibodies in TcVac2-vaccinated/challenged mice was in the order of TcG4>TcG2>TcG1. Mice vaccinated with cytokine adjuvants exhibited slightly higher levels of parasite- and TcG4-specific antibody response than the controls given vector only, thus, indicating that cytokines alone moderately enhanced the antibody response upon challenge infection (Fig. 2). No significant difference was detected in the level of parasite-induced IgG2a in vaccinated and non-vaccinated mice, and in mice given cytokine adjuvants only (data not shown). Together, these data showed that TcVac2 vaccination of mice resulted in a rapid expansion of antibody response against challenge *T. cruzi* infection, and this antibody response was maintained at least up to day 120 pi when animals were sacrificed.

**Figure 3:**
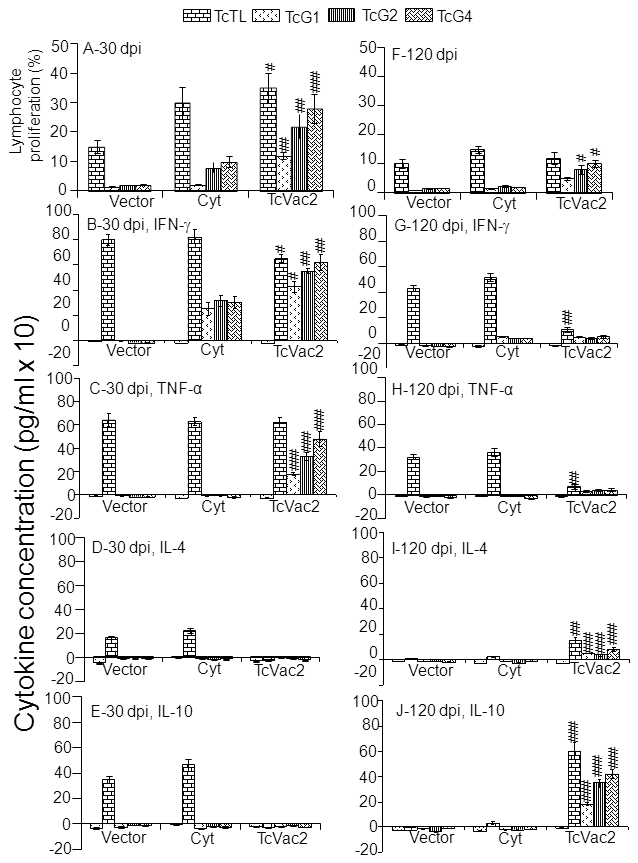
TcVac2-immunized mice exhibited significant increase in lymphocyte proliferation and type 1 cytokine production in response to acute *T. cruzi* infection. Spleen cells from mice sacrificed at day 30 (**A–E**) and 120 (**F–J**) pi were *in vitro* stimulated with TcTL or recombinant antigens for 48 h. The extent of lymphocyte proliferation was determined by an MTT assay (**A&F**). The IFN-γ (**B&G**), TNF-α (**C&H**), IL-4 (**D&I**), and IL-10 (**E&J**) levels in cell-free supernatants were measured by an ELISA (n = 8 mice/group, ^#^ *p*<0.05, ^##^ *p*<0.01,^ ###^ *p*<0.001).

**Figure 4 pntd-0000797-g004:**
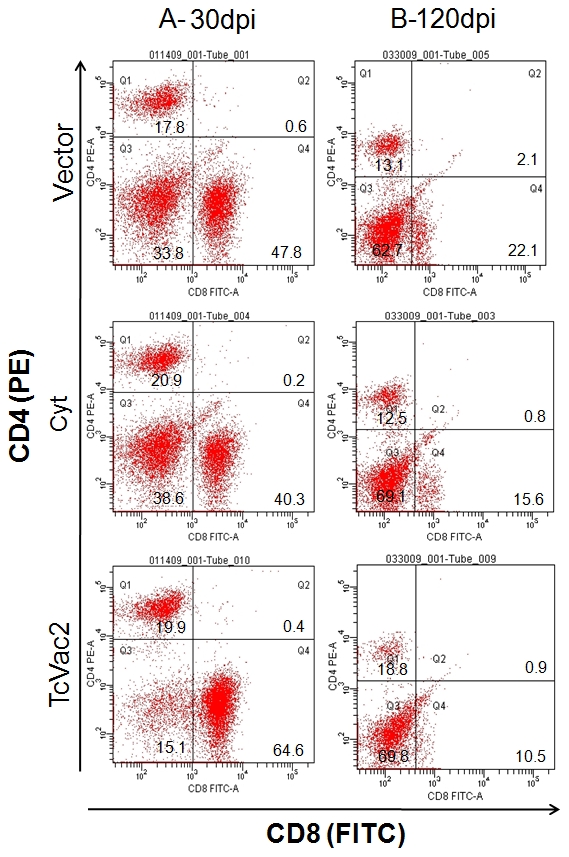
CD8^+^ T cell subset dominated in splenic T cell population of TcVac2-vaccinated/acutely-infected mice. Mice were vaccinated and challenged with *T. cruzi,* and harvested at day 30 (**A**) and 120 (**B**) post-infection, corresponding to acute infection phase and chronic disease phase, respectively. Spleen cells were incubated for 30 min with FITC-conjugated anti-CD8 and PE-conjugated anti-CD4 antibodies and CD4^+^ and CD8^+^ T cell subsets monitored by flow cytometry.

Splenocytes harvested at day 30 and 120 pi were *in vitro* stimulated with TcTL or recombinant antigens, and we measured the lymphocyte proliferation by MTT assay and cytokine levels by ELISA (Fig. 3). All mice, irrespective of vaccination regimen, responded to acute infection by a strong increase in parasite-specific lymphocyte proliferation (Fig. 3A), and IFN-γ (Fig. 3B) and TNF-α (Fig. 3C) production (note the brick-bars with Tc-lysate stimulation). However, a potent increase in antigen-specific lymphocyte proliferation, and IFN-γ and TNF-α production (TcG4>TcG2>TcG1) was primarily observed in vaccinated/infected mice (Fig. 3A–C, p<.05-p<0.001). Further, vaccinated/acutely-infected mice mounted a type 1 biased cytokine response as was evidenced by undetectable levels of IL-4 (Fig. 3D) and IL-10 (Fig. 3E) cytokines in supernatants of splenocytes stimulated with TcTL or specific antigens. In comparison, mice injected with vector or cytokines only exhibited a mixed response and mounted TcTL-dependent production of IL-4 (165**±**11.6 and 220**±**18.2 pg/ml, respectively) and IL-10 (350**±**25.9 and 465**±**45.5 pg/ml, respectively) cytokines (Fig. 3D&E). The mean percentage of CD4^+^ and CD8^+^ T cells in acutely-infected mice given vector alone, cytokines only, and TcVac2 vaccine were 17.8**±**1.4, 21.9**±**2.32, and 20.8**±**1.7; and 47.8**±**3.5, 42.3**±**3.7, and 65.5**±**4.2, respectively (Fig. 4A). Together these data showed that immunization with TcVac2 led to induction of a strong CD8^+^ T cell and type 1 biased cytokine response to acute infection by *T. cruzi*. In absence of vaccine-mediated polarization, mice elicited a mixed type 1/type 2 cytokine response against *T. cruzi* infection.

In chronic phase (120 dpi), all infected mice, irrespective of vaccination status, exhibited an overall decline in the extent of lymphocyte activation and proliferation (Fig. 3F) as compared to what was noted in acute stage (Fig. 3A). Yet, vaccinated/chronically-infected mice exhibited a switch to CD4^+^ T cells and type 2 cytokines. This was evident by the fact that as compared to vaccinated/acutely-infected mice, spleen cells of vaccinated/chronic mice consisted a significantly higher number of CD4^+^ (18.8%) than CD8^+^ (10.5%) T cells (Fig. 4B) that produced higher levels of IL-4 (Fig. 3I) and IL-10 (Fig. 3J) cytokines, and decreased levels of IFN-γ (Fig. 3G) and TNF-α (Fig. 3H) cytokines (IFN-γ+TNF-α<IL-4+IL-10). This polarization of cytokine response (type 2>type1) in vaccinated/chronically infected mice was evident when splenocytes were stimulated with Tc-lysate or TcG1, TcG2 and TcG4 antigens (Fig. 3G–J). In comparison, mice injected with vector alone or cytokines only maintained a pro-inflammatory milieu during chronic phase as was evidenced by higher mean percentage of CD8^+^ (22.1 and 15.6%, respectively) than CD4^+^ (15.5and 11.5%, respectively) T cells. Further, chronically infected mice injected with vector alone or cytokines only exhibited higher levels of TcTL-stimulated splenic release of IFN-γ (435**±**22.8 and 520**±**32.5 pg/ml, respectively, Fig. 3G) and TNF-α (325**±**25 and 365**±**34.2 pg/ml, respectively, Fig. 3H) cytokines; and low-to-none IL-4 (Fig. 3I) and IL-10 (Fig. 3J) cytokines. These data suggested that mice vaccinated with TcVac2 were equipped to control the chronic activation of CD8^+^ T cell proliferation and proinflammatory cytokine (IFN-γ and TNF-α) production that are considered pathological in Chagas disease.

**Figure 5:**
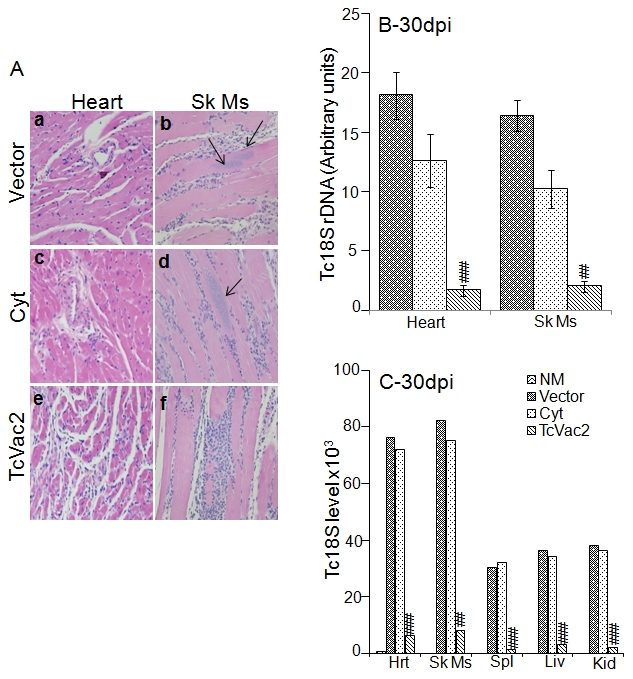
Enhanced infiltration of inflammatory infiltrate contributed to control of acute tissue parasite burden in TcVac2-immunized mice. Mice were vaccinated with TcVac2 or given vector or cytokines (cyt) alone, and challenged with *T. cruzi*. (**A**) Shown are H&E staining (blue-nuclear and pink-muscle/cytoplasm/keratin) of heart tissue and skeletal muscle (Sk Ms) sections at day 30 pi (magnification: 20X). Arrows mark parasite nests in panels b & d. (**B&C**) Total DNA from Heart (Hrt), Skeletal Muscle (Sk Ms), Spleen (Spl), Liver (Liv) and Kidney (Kid) was isolated and used as a template for the amplification of *T. cruzi* 18SrDNA sequence by traditional PCR (**B**) or real time PCR (**C**). Standard deviation was <12% for the data presented in the Figure 5C (^##^ *p*<0.01, ^###^ *p*<0.001).

Histological analysis revealed extensive infiltration of inflammatory cells in the heart and skeletal muscle of all acutely-infected mice (Fig. 5A). In agreement with enhanced activation of B and T cell responses, the extent of inflammatory infiltrate in heart tissue and skeletal muscle of vaccinated/acutely-infected mice (histological score 2–4, Fig. 5Ae-f) was significantly higher than that detected in acutely-infected mice injected with vector or cytokines only (histological score 1–3) (Fig. 5Aa–d). Accordingly, tissue parasite foci were remarkably reduced (0–2 per microscopic field, mf) in TcVac2-vaccinated mice harvested at day 30 pi (Fig. 5Ae-f). In comparison, mice injected with vector only (2–10 parasite nests/mf, Fig. 5Aa-b) or cytokine adjuvants (1–5 parasite nests/mf, Fig. 5Ac-d) failed to control acute tissue parasite burden. The decline in tissue parasite burden in vaccinated/acutely-infected mice was validated by a semi-quantitative PCR (Fig. 5B) and real time PCR (Fig. 5C). Our data demonstrated a 89–97% reduction in amplification of *Tc18SrDNA* sequence in heart (91.6%), skeletal muscle (89.3%), spleen (96.8%), liver (91%) and kidney (94%) of vaccinated/acutely-infected mice when compared to that detected in infected mice that were injected with vector or cytokines only (Fig. 5B-5C, *p*<0.001).

**Figure 6:**
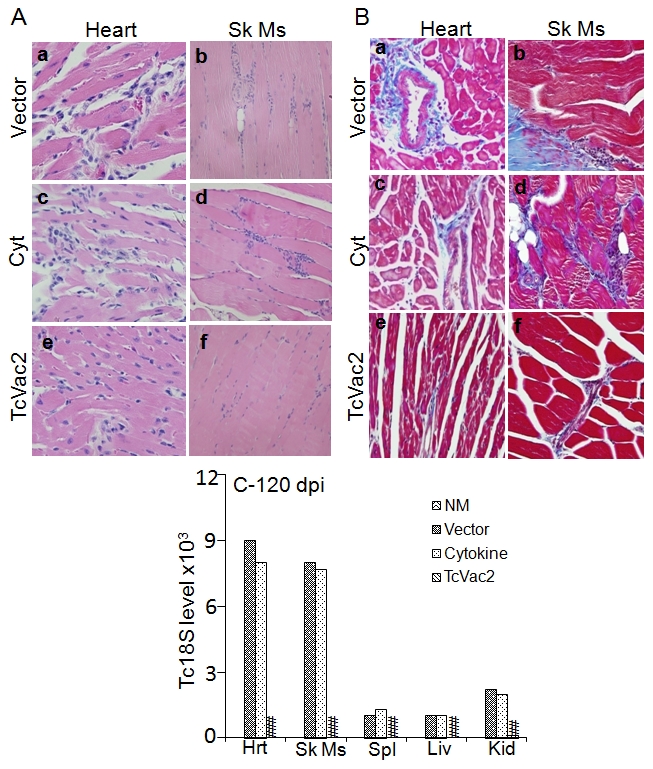
Chronic inflammatory infiltrate, tissue fibrosis and parasite burden were arrested inTcVac2-immunized/infected mice. Heart tissue and skeletal muscle were harvested at 120 dpi (chronic phase) and subjected to H&E (**A**) or Masson's Trichrome (**B**) staining (magnification: 20X). The intense blue color (Fig. 6B) shows the collagen deposition (fibrotic area). (C) Real time PCR amplification of *T. cruzi* 18SrDNA (as in Fig. 5C).

At the chronic stage, the inflammatory infiltrate (Fig. 6Ae-f) and tissue fibrosis (Fig. 6Be-f) were particularly absent in heart tissue and skeletal muscle of TcVac2-vaccinated mice (histological score 0–1). Real time PCR amplification of parasite-specific Tc18SrDNA sequence (for 50 cycles) detected no signal in the heart, skeletal muscle, spleen, and liver tissue vaccinated/chronic mice and Tc18S signal in kidney of vaccinated/chronic mice was reduced by >95% as compared to that noted in non-vaccinated/chronic mice (Fig. 6C). In comparison, extensive-to-moderate levels of inflammation (histological score 2–4, Fig. 6Aa–d), tissue fibrosis (histological score 2–4, Fig. 6Ba–d), and persistence of parasites (Fig. 6C) dominated in heart and skeletal muscle of chronic mice injected with vector or cytokines only. Noticeable larger blue-stained areas, indicator of fibrosis, were evident in Masson-trichrome-stained heart and skeletal muscle sections of chronically-infected mice that were given vector or cytokines only (Fig. 6Ba–d) as compared to TcVac2-immunized mice (Fig. 6Be-f). These results demonstrated that TcVac2 was effective in arresting the parasite persistence and associated inflammatory pathology and tissue fibrosis in chronic Chagasic mice.

## Discussion

The present study was performed to test the protective efficacy of TcVac2 vaccine. The antigenic candidates included in TcVac2 were identified by computational analysis of *T. cruzi* sequence database and selected because they were conserved among several clinically relevant *T. cruzi* strains, and expressed as plasma surface proteins in infective trypomastigote and intracellular amastigote stages. The TcG1-, TcG2- and TcG4*-*specific antibodies exhibited lytic activity against the infective trypomastigote form, suggesting their potential utility as vaccine candidates [26]. We chose DNA-prime/protein-boost approach to deliver the subunit vaccine because DNA vaccines induce a T cell response that can be strongly boosted by recombinant (or antigenically similar) proteins, and DNA/protein vaccines have been shown to elicit stronger, long-term immunity against intracellular pathogens than is noted by DNA/DNA vaccines [31], [32] [38]. We utilized IL-12 and GM-CSF expression plasmids as adjuvants with DNA-prime dose as these cytokines induce type 1 B and T cell responses [39], [40], and are shown to significantly enhance the protective immunity elicited by the vaccine candidates in mice [5]. To the best of our knowledge, no published report has tested the protective efficacy against *T. cruzi* of a heterologous prime-boost vaccine constituted of the selected candidates, and this is the first report demonstrating vaccine's efficacy in reducing the tissue parasite burden below detection limits in chronic chagasic animals.

Mice immunized with TcVac2 elicited a strong parasite-specific antibody response that was rapidly expanded after challenge *T. cruzi* infection. The levels of IgG2b and IgG1 antibody isotypes were significantly elevated in TcVac2-vaccinated mice, and the antibody response was primarily of the Th1 type with IgG2b/IgG1 ratios being >1 (Fig. 1). Others have shown that the lytic IgG1 and IgG2 antibodies are involved in parasite clearance [41], [42]. For example, IgG2a elicited by immunization with kinetoplastid membrane protein-11-encoding plasmid controlled *T. cruzi* infection in rodents [11], while IgG1 and IgG2b were found to be predominant isotypes in mice immunized with complement-regulatory protein and trans-sialidase antigens [10], [43]. Another study showed low-to-none IgG1 levels in chagasic dogs exhibiting cardiomegaly and chronic pathology [44]. Previously, we have shown that TcG1-, TcG2- and TcG4-specific antibodies were lytic in nature and efficiently killed trypomastigotes in a complement-dependent manner [26]. In this study, TcVac2-vaccinated mice exhibited a better control of *T. cruzi* infection than was observed in mice injected with vector alone or cytokines only, and this protection was correlated with persistence of a high ratio of IgG2b/IgG1 antibodies (Fig. 2). The tissue parasite foci and parasite burden were significantly reduced in TcVac2-immunized mice at the acute stage (Fig. 5C). Importantly, parasites were undetectable by a sensitive real time PCR approach in multiple organs (heart, skeletal muscle, liver, spleen) and barely detectable in kidney of vaccinated mice at chronic stage (Fig. 6C). We surmise that vaccination with TcVac2 resulted in a rapid expansion of antigen-specific Th1 antibody response upon challenge infection that contributed to effective control of acute parasite burden and led to undetectable parasite persistence in chronic stage.

A collection of studies in knock-out mice or in rodents treated with antibodies to deplete specific immune molecules have shown that beside *T. cruzi-*specific lytic antibody response, an efficient control of acute parasitemia also requires concerted activities of macrophages, Th1 helper cells, and cytotoxic T lymphocytes (CTLs) (reviewed in [45]). IFN-γ and TNF-α cytokines are believed to be the key inducers of macrophage activation, and nitric oxide (NO) production and oxidative burst are required for parasite clearance in early stages of infection [46]. CD4^+^ T cells secreting type 1 cytokines and CD8^+^ CTLs capable of killing the infected cells have been implicated in control of intracellular *T. cruzi* [47], [48]. The role of Th1 cytokines in the immune control of *T. cruzi* has been addressed by the studies demonstrating that over production of type 2 cytokines or blockage of type 1 cytokine production correlates with increased susceptibility to *T. cruzi* infection [49]–[52]. In this study, analysis of splenocyte T cell subsets, and cytokine production in TcVac2-vaccinated/infected mice showed a polarized response with dominance of IFN-γ, TNF-α, and CD8^+^ T cells in the acute phase, and IL-4, IL-10, and CD4^+^ T cells in the chronic phase (Figs. 3,4). Mice given vector alone or cytokines only elicited mixed type 1/type 2 responses inefficient at achieving parasite clearance (Figs. 3–5). We propose that polarization of immune response in vaccinated mice was beneficial as protective effect of CD8^+^ T cells and type 1 cytokines (IFN-γ and TNF-α) was not suppressed by IL-4 and IL-10 cytokines leading to an effective control of acute infection (Fig. 5). A switch to type 2 dominance in vaccinated/chronic mice likely suppressed the infiltration of inflammatory infiltrate that otherwise was exacerbated (Fig. 6) with sustained IFN-γ production in non-vaccinated/chronic mice. The efficacy of heterologous DNA-prime/protein-boost TcVac2 against *T. cruzi* was highlighted by the fact that DNA-prime/DNA-boost vaccines have not been very successful in providing sterile immunity to *T. cruzi* [20].

The persistence of inflammatory responses associated with tissue fibrosis and cell death are hallmarks of chronic Chagas disease. It is suggested that CD8^+^ T cells, the dominant resident immune cells in the heart have a toxic effect on the host, as many of the CD8^+^ T cells express granzyme A that causes non-specific bystander cell death and tissue necrosis [53]. Others have shown that the frequency of *T. cruzi*-specific IFN-γ^+^ T cells correlate with severity of chronic disease in chagasi*c* humans and experimental animals [54], [55]. Circulating levels of IFN-γ and its production by PBMCs *in vitro* stimulated with *T. cruzi* antigens has been implied a*s* a biomarker in identifying asymptomatic, seropositive patients at risk of developing symptomatic chronic cardiomyopathy [54]. In this study, it was particularly interesting to note that frequency of activated CD8^+^ T cells and IFN-γ and TNF-α cytokine levels in lymphoid of TcVac2-vaccinated/chronic mice were considerably lower than that observed in unvaccinated/chronic mice (Figs. 3, 4). Further, heart and skeletal muscle of vaccinated mice exhibited minimal-to-no infiltration of inflammatory infiltrate. Also parasite 18SrDNA-specific real time PCR signal was minimal or not amplified in the tissues (heart, skeletal muscle, spleen liver and kidney) during the chronic stage (Fig. 6). Based on these studies and our data, we conclude that TcVac2 elicited effective immune responses capable of controlling the acute parasite burden below a threshold level, and subsequently, prevented the consistent activation and infiltration of inflammatory cells in the heart that otherwise occur due to parasite persistence and result in tissue destruction during the chronic phase.

In summary, we have demonstrated that DNA-prime/protein-boost TcVac2 vaccine provided resistance to *T. cruzi* infection. Mice immunized with TcVac2 responded to *T. cruzi* infection with a rapid and potent expansion of type 1 antibodies, CD8^+^ T cells, and proinflammatory cytokines that effectively controlled the acute parasitemia and tissue parasite burden. Consequently, evolution of chronic immunopathology and tissue damage, an outcome of parasite persistence and consistent immune activation, were absent in vaccinated mice.
